# Banhabaekchulchunma-tang and chongsanggeontong-tang (herbal medicine) effect on migraine: A protocol for a systematic review of controlled trials

**DOI:** 10.1097/MD.0000000000010119

**Published:** 2018-03-16

**Authors:** Jihye Seo, Cheol hyun Kim, Hongmin Chu, Yeonju Moon, Sangkwan Lee

**Affiliations:** a Clinical Trial Center, Wonkwang University Gwangju Hospital, Gwangju; b Department of Internal Medicine, College of Korean Medicine, Wonkwang University, Iksan, Jeonbuk, Republic of Korea.

**Keywords:** Banhabaekchulchunma-tang, Chongsanggeontong-tang, migraine, systematic review

## Abstract

**Background:**

Migraine is a common disease of primary headache, which it causes many kinds of secondary diseases or symptoms. The treatment drugs have sometimes shown side effects such as overuse headache, stroke, and cardiovascular disease. Therefore, the demand for complementary and alternative interventions for migraine has increased. Herbal medicines are a representative intervention to treat migraine traditionally. Among these herbal medicines, Banhabaekchulchunma-tang (BBT) and Chongsanggeontong-tang (CGT) have been most commonly used for migraine treatment in traditional clinical practice. However, to our knowledge, there has been no systematic review considering the efficacy and safety of BBT or CGT on migraine. This protocol aims to perform a systematic review for assessing the effectiveness and safety of BBT or CGT on Migraine.

**Methods:**

This protocol was developed according to the guidelines outlined in the Preferred Reporting Items for Systematic Reviews and Meta-Analysis Protocol (PRISMA-P) and registered on the international prospective register of systematic reviews (PROSPERO). The randomized controlled clinical trials of BBT or CGT for migraine treatment will be searched in following 8 databases from their inception to December 2016: Medline, EMBASE, the Cochrane Central Register of Controlled Trials, OASIS, the Korean Traditional Knowledge Portal, the Korean Medical Database, DBPIA, and the China National Knowledge Infrastructure. Study selection, data extraction, assessment with risk of bias, and data analysis will be performed in order. In this study, headache pain intensity and the total treatment effective rate will be evaluated as primary outcomes.

**Results and Conclusion:**

we propose the current protocol to evaluate the effectiveness and safety of BBT or CGT for migraine systematically.

**Ethics and dissemination:**

This systematic review will not need ethical approval, because it does not involve human beings. We will publish this systematic review electronically in a peer-reviewed journal.

**Systematic review registration:**

PROSPERO CRD42018076171 for BBT and CRD42018085130 for CGT

## 1 Introduction

Migraine is a primary headache characterized by recurrent episodes of moderate-to-severe pulsating headache, mostly unilateral, with nausea, photophobia, or phonophobia, which have negative impact on patients’ quality of life.^[1,2]^ Migraine has been reported to be a risk factor for stroke, cervical artery dissection, and structural changes in the brain.^[3–5]^ Therefore, optimal migraine interventions may help patients’ well-being and to prevent secondary diseases caused by migraine.

Although there are some drugs for migraine treatment such as nonsteroidal anti-inflammatory drugs, triptans, and ergotamines, these drugs have often shown the side effects.^[6]^ For example, triptans should be avoided in patients with cardiovascular disease or stroke, because they can narrow arteries.^[6,7]^ When triptans is also overused, it might cause medication overuse headache.^[8]^ The main side effects of ergotamines are myocardial infarction, ischemia of limb extremities, fibrotic changes, and overuse headache.^[9]^ For these reasons, demand for complementary and alternative medicine therapies have increased.

Herbal medicine, acupuncture, moxibustion, and cupping for migraine treatment have been traditionally used in East. So, these traditional therapies can be a candidate for migraine treatment.^[10]^ Banhabaekchulchunma-tang (BBT) and Chongsanggeontong-tang (CGT) are commonly herbal medicines which have been traditionally used for migraine.^[11]^ Ou Jianghong et al^[12]^ reported that BBT showed the treatment effect on migraine. In addition, some studies also showed that CGT was effective on pain, convulsion, hyperlipidemia, and chronic tension-type headache.^[13–15]^ However, to our knowledge, there has been no systematic review on the effectiveness of BBT or CGT on migraine. Therefore, we propose the current protocol to evaluate the effectiveness and safety of BBT or CGT for migraine systematically.

## 2 Methods and analysis

The protocol for this systematic review has been registered on PROSPERO with registration number (CRD42018076171 and CRD42018085130). This protocol follows the guidelines outlined in the Preferred Reporting Items for Systematic Reviews and Meta-Analysis Protocol (PRISMA-P) and the Cochrane Handbook for Systematic Reviews of Interventions.^[16]^ If we need to change this protocol, the changes would be described in our full review.

### 2.1 Data sources and search strategy

The following databases including 4 Korean medical databases and 1 Chinese database will be electronically searched from their inception to December 2016: Medline, EMBASE, the Cochrane Central Register of Controlled Trials), OASIS, the Korean Traditional Knowledge Portal, the Korean Medical Database, and DBPIA, the China National Knowledge Infrastructure.

To perform a comprehensive and focused search, experienced systematic review researchers will help to develop a search strategy. The searched terms are as follows: migraine disorders, headache, migraine attack, episodic migraine, herbal medicine, traditional Korean medicine, Kampo medicine, and traditional Chinese medicine. In addition, we will more search Banhabaekchulchunma-tang or Banxia Baizhu Tianma Decoction or Chongsanggeontong-tang in Korean and Chinese databases. An example of search strategy for Medline database shown in Table 1 will be modified and used for the other databases.

**Table 1:**
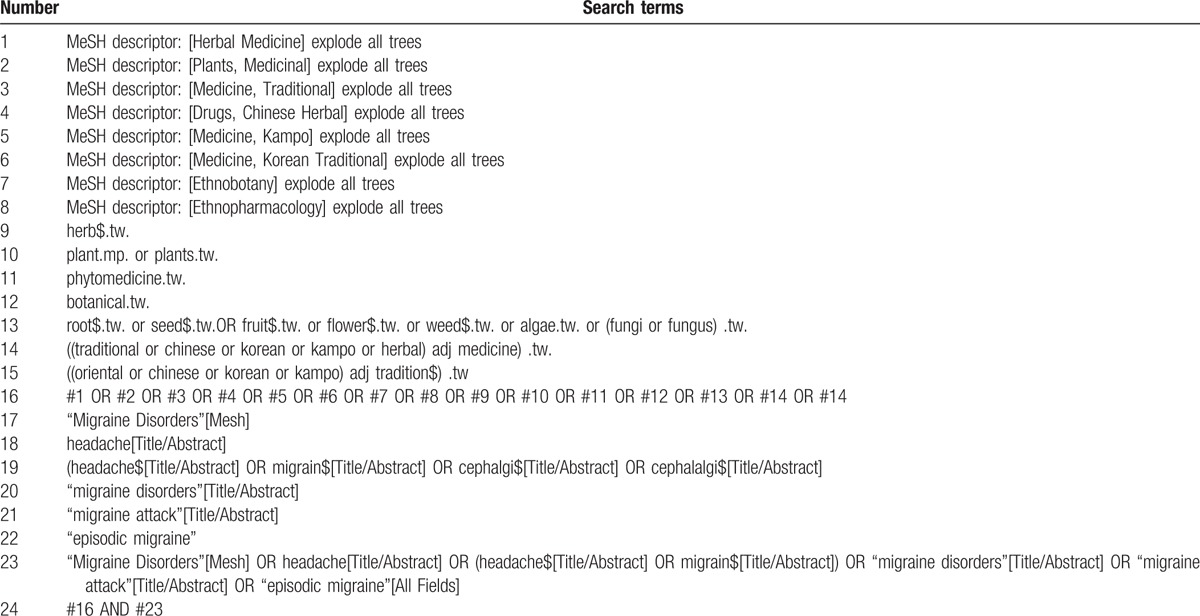
Searching strategy for Medline.

### 2.2 Inclusion criteria

#### 2.2.1 Types of participants and interventions

The diagnostic criteria will be developed according to the International Classification of Headache Disorders. We will include participants diagnosed as migraine, regardless of gender or age, according to the diagnostic criteria. Interventions to be reviewed are BBT or CGT alone or combinations with other interventions to treat the migraine. When BBT or CGT used as combinations with other treatments, the control group should also receive the same combination treatments.

#### 2.2.2 Types of studies

This review will include randomized controlled clinical trials (RCTs) and quasi-randomized controlled trials. Controlled (nonrandomized) clinical trials, case series, and case reports will be excluded. The studies will be limited to the full text articles written in English, Korean, or Chinese.

#### 2.2.3 Types of outcomes

Primary outcomes are as follows:

(1) *Headache pain intensity*: Headache pain intensity is indicated on the visual analogue scale.
(2) *The total treatment effective rate*: The total treatment effective rate is calculated by the number of patients with improvement in the number of those with migraine attacks.

Secondary outcomes are as follows:

(1) Impact of migraine-related symptoms (headache frequency, headache duration time) as measured with validated questionnaires
(2) Migraine-associated symptoms (nausea, photophobia, phonophobia)
(3) Quality of life: evaluated by general or migraine-specific scales
(4) Adverse events

### 2.3 Study selection

At the end of the search process, the results are exported to the referencing software (Endnote version 6, Thomas Reuters). First, 2 independent researchers evaluate and select the titles and abstracts of studies, and then evaluate the full text of the selected studies and confirm eligibility of them for our review. Disagreements in selection and evaluation are resolved by involved researchers’ discussion. Using the PRISMA compliant flow chart (http://www.prisma-statement.org), study screening and selection process are documented and summarized. In this flow chart, the excluding reasons are provided.

### 2.4 Data extraction and quality assessment

Data extraction and quality assessment of studies are performed and cross-checked by 2 independent researchers. Disagreements of the results between 2 researchers are resolved through researchers’ discussion. If the disagreement cannot be resolved by discussion, it would be resolved by the arbiter. When the selected studies that do not have sufficient data, we will e-mail the authors to request the data whenever possible.

To perform data extraction and quality evaluation of RCT, we use data extraction form (Excel) designed by all researchers’ consensus. The extracted data will include the first author, year of publication, patient characteristics, intervention and comparison details, sample size and dropouts, outcomes, and adverse events.

The quality assessment is performed using the risk of bias tool from the Cochrane Handbook V.5.1.0. The risk of bias tool includes random sequence generation, allocation concealment, blinding of the participants and personnel, blinding of the outcome assessments, incomplete outcome data, selective reporting, and other sources of bias.

### 2.5 Data analysis

To compare the outcomes of the studies, we perform the data analysis between the intervention and control groups. For quantitative synthesis, we count a single measurement only once for each outcome of each participant. Each participant's outcome are counted or measured only once. The data analysis and synthesis are performed using the RevMan (the Cochrane Collaboration's software program Review Manager Version .5.2.7 for Windows).

For dichotomous or continuous data, we present the outcomes as relative risks with 95% confidence intervals (CIs) or use the mean differences with 95% CIs to calculate the effect size of the interventions, respectively. For the outcome values on different scales, we calculate using the standard mean difference. To perform the meta-analysis, we pool the data using fixed or random effects model considering the type of outcomes. When data quantitative synthesis is not possible, we will analysis the data qualitatively.

To evaluate quality of evidence, reviewers use the Grading of Recommendations Assessment, Development and Evaluation approach.

#### 2.5.1 Assessment of heterogeneity and reporting biases

The heterogeneity between studies is assessed by the *I*^2^ statistic value calculated with the RevMan program and visually inspecting the forest plots. *I*^2^ > 50 are considered to mean the high heterogeneity. In the case of high heterogeneity, we investigate the possible causes of the heterogeneity and perform subgroup analyses based on different combinations of intervention with other treatments, or to the other factors affecting the outcomes.

We assess the reporting biases with funnel plots when sufficient studies (at least 10 studies) are available. When there are the reporting biases, we will attempt to explain the possible reasons for any asymmetries in reporting.

## 3 Discussion

Managing migraine is important because migraine has negative impact on patients’ life and causes many secondary diseases.^[4]^ Many drugs have been used for migraine treatment, some drugs have often shown side effects such as overuse headache, stroke, and cardiovascular disease.^[8,9]^ Therefore, demand for complementary and alternative therapies for migraine treatment have increased.

BBT and CGT for migraine treatment have been traditionally used in East.^[10,11]^ In traditional Chinese medicine, it is thought that phlegm-retained fluid can cause migraine.^[11]^ BBT is one of the herbal medicines to remove phlegm-retained fluid in the body.^[11]^ There are also CGT RCT and several case reports for chronic tension-type headache and migraine, respectively.^[15,17]^ However, to our knowledge, there has been no systematic review considering the safety and effectiveness of BBT or CGT on migraine.

Therefore, we developed a protocol to assess the effectiveness and safety of BBT and CGT on migraine systematically. However, this protocol may have some limitations such as the limited language trials, different dosage of herbal medicine and age of patients, and small sample of included trials, which may lead to bias.

## 4 Contributors

CK, JS, and YM conceived and drafted the protocol. SL and HC planned and revised the protocol and the manuscript. SL and HC examined the protocol in the view of clinician. All authors have approved the final manuscript.
